# Dose-Response Effects of a Multimodal Physical Activity Intervention on Selective Attention in Schoolchildren from Bogotá

**DOI:** 10.3390/children13030364

**Published:** 2026-03-04

**Authors:** Jaime Alvarado-Melo, Henry León-Ariza, Angela Figueroa-Palacios, Adriana Gutiérrez-Galvis, Manuel Riveros-Medina, Andrés Rosa-Guillamon, Eliseo Garcia-Canto

**Affiliations:** 1 Universidad de La Sabana, Chía 250001, Colombia; henry.leon@clinicaunisabana.edu.co; 2 Facultad de Educación, Universidad Libre, Bogotá 111071, Colombia; angelam-figueroap@unilibre.edu.co (A.F.-P.); manuel.riverosm@unilibre.edu.co (M.R.-M.); 3 Escuela Militar de Cadetes General José María Córdova, Área Deportes, Bogotá 111211, Colombia; 4 Universidad Nacional de Colombia, Bogotá 111321, Colombia; argutierrezg@unal.edu.co; 5 Facultad de Educación, Universidad de Murcia, 30003 Murcia, Spain; andres.rosa@um.es (A.R.-G.); eliseo.garcia@um.es (E.G.-C.)

**Keywords:** selective attention, physical activity, active breaks, schoolchildren

## Abstract

**Highlights:**

**What are the main findings?**

- An 8-week multimodal intervention improves selective attention in youth, irrespective of baseline physical activity levels.

**What are the implications of the main findings?**

- Active classroom breaks represent a low-cost and efficient strategy to enhance selective attention without requiring additional sports infrastructure.

**Abstract:**

**Background/Objectives**: The dose–response relationship between physical activity (PA) and cognitive indicators such as selective attention remains a subject of debate among professionals in education and neuroscience. The aim of this study was to determine the effects of an 8-week multimodal intervention program on selective attention in schoolchildren from Bogotá, Colombia, by comparing different intensities and practice contexts. **Methods**: A quasi-experimental study was conducted with 267 students aged 8–14 years, divided into four groups: control group (CG *n* = 69), light physical activity/active breaks (LPA; *n* = 64), moderate physical activity (MPA; *n* = 66) and moderate-to-vigorous physical activity (MVPA; *n* = 68). The intervention was delivered as follows: the control group followed usual school routines; LPA through classroom active breaks (10 min, 3×/day, 3 days/week); MPA during school recess (30 min, 2×/week), and MVPA during physical education classes (90 min, 1×/week). Selective attention was assessed using the d2-R test. Linear Mixed Models (LMMs) were applied to analyze the Group × Time interaction while accounting for the hierarchical structure of the data (students nested within eight schools). **Conclusions**: Moderate-to-vigorous physical activity (Δ = +17.49) and light-intensity active breaks (Δ = +15.47) were effective in strengthening selective attention. These findings suggest that school-based physical activity should extend beyond physical education classes and incorporate movement-based activities within the classroom.

## 1. Introduction

Globally, physical inactivity among children and adolescents has reached alarming levels and has become a growing concern for government agencies, which have sought measures to mitigate its effects on health. The World Health Organization, in its guidelines on physical activity (PA) and sedentary behavior, suggests that to obtain significant health benefits, children and adolescents aged 5–17 years should accumulate at least 60 min per day of moderate-to-vigorous physical activity [1]. However, reports such as the Global Matrix 4.0, published by the Global Alliance for Active Healthy Children, which assessed 57 countries, indicate that physical activity levels worldwide remain alarmingly low [2]. In Colombia, the Report Card on Physical Activity for Children and Adolescents indicates that fewer than half of the school-aged population meet these recommended standards [3]. Paradoxically, the school context has become a setting characterized by a high prevalence of sedentary behaviors, where academic processes prioritize activities that require prolonged sitting during the school day [4,5]. This not only affects metabolic health but also limits the opportunities that physical activity offers for neurocognitive development in schoolchildren [6]. The recent scientific literature has established a positive association between PA and physical fitness levels and the enhancement of several cognitive functions, including memory, attention, and cognitive flexibility [7,8,9,10].

Selective attention, defined as an individual’s ability to focus on relevant stimuli while filtering out irrelevant information [11], is fundamental for academic success. Physical activity has been associated with neurophysiological changes, including increased cerebral blood flow and the release of brain-derived neurotrophic factor (BDNF), which facilitates synaptic plasticity and may enhance attentional control [12,13].

Despite the extensive empirical evidence and academic consensus regarding the general benefits of physical activity on cognitive functions, a methodological gap remains concerning the “dose” of physical activity, expressed in terms of the optimal intensity and volume of movement. In a previous systematic review conducted by our research group, it was identified that the heterogeneity in intervention intensities, durations, and contexts does not allow the establishment of precise guidelines for physical activity practice in school settings [14]. It remains unclear whether the benefits observed in cognitive functions such as selective attention are exclusively the result of activities involving vigorous intensities or whether light-intensity activities, such as active breaks, may also produce comparable or even greater effects on attention, particularly selective attention.

In light of the above, and in an effort to address this gap, the present study describes a physical activity intervention program implemented across different intensities and school contexts over an eight-week period in eight educational institutions in Bogotá, Colombia (*n* = 267 children and adolescents aged 8–14 years). Unlike previous studies that focused their interventions on a single PA modality, this study compared the simultaneous implementation of four intervention groups with different PA modalities, with each group comprising two schools.

The control group (CG; *n* = 69) received no intervention. The light-intensity physical activity group (LPA; *n* = 64) participated in active classroom breaks. The moderate-intensity physical activity group (MPA; *n* = 66) engaged in physical activity implemented during school recess. The moderate-to-vigorous physical activity group (MVPA; *n* = 68) performed physical activity during physical education classes.

The objectives of this study were, first, to empirically evaluate the effect of PA on the enhancement of selective attention and, second, to determine the differential effects of each PA modality on selective attention capacity using the d2-R test. This was conducted under a pretest–posttest design and analyzed using linear mixed models (LMMs), which account for the inherent variability across school-based intervention groups.

Our hypothesis was that a higher intensity of physical activity would produce greater improvements in selective attention, so the MVPA group would show the highest gains compared to the other groups.

## 2. Materials and Methods

### 2.1. Study Design

**Figure 1:**
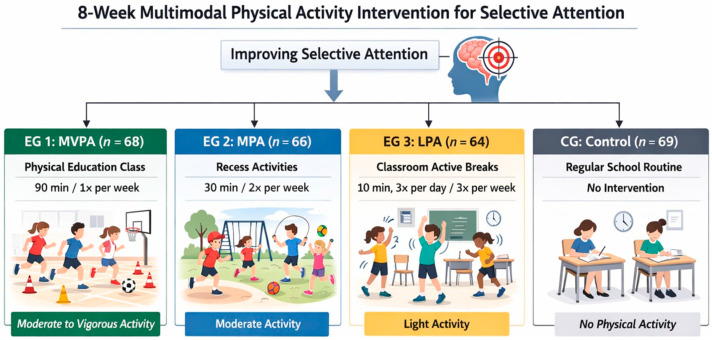
Study design and intervention groups.

This study employed a quasi-experimental approach with a pretest–posttest design, including an eight-week intervention and four simultaneously implemented intervention groups. This approach allowed for the comparison of three (experimental groups—EGs) PA modalities with different volumes, school contexts, and intensities (MVPA, MPA, and LPA) against a control group (CG) that maintained traditional school routines. To ensure the internal validity of this study and minimize the risk of bias, a two-step process was implemented. First, the eight schools participating in this study were selected through convenience sampling. Second, a sealed-envelope randomization procedure, conducted by an independent researcher, was used to assign each school to one of the groups. The structure and dose of the intervention for each group are described in Figure 1.

### 2.2. Participants

**Figure 2:**
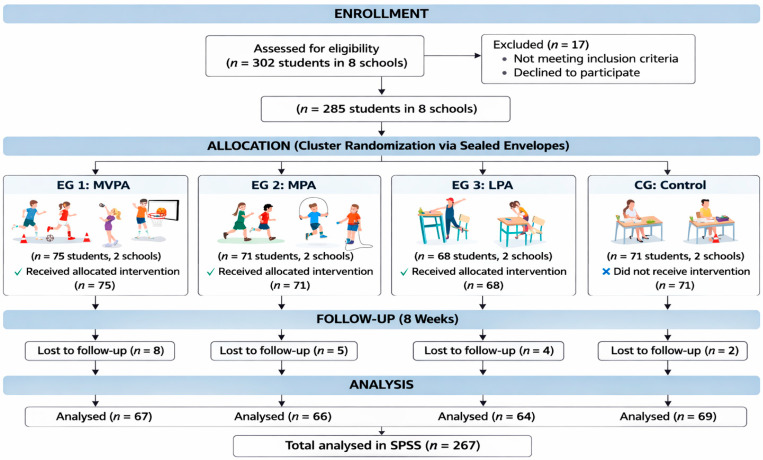
Participant flow diagram.

Children and adolescents (*n* = 267; 113 girls and 154 boys) who participated in this study were recruited from eight schools with diverse socioeconomic backgrounds in Bogotá, Colombia, and were aged between 8 and 14 years. The following inclusion and exclusion criteria were applied: regular school attendance; provision of written parental informed consent and child assent; absence of a diagnosis of attention deficit hyperactivity disorder; absence of medical recommendations contraindicating physical activity; and absence of diagnosed severe learning disorders. Figure 2 illustrates the enrollment and retention process of the study population throughout the eight-week intervention period.

### 2.3. Procedures

**Figure 3:**
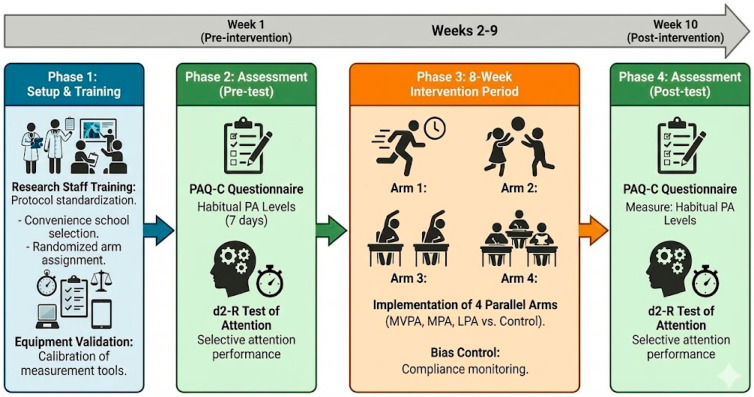
Experimental procedure timeline.

The development of this study was organized into four phases, which are described in Figure 3. This study began with the development of the study protocol, the assessment of potential methodological risks of bias, the training of research assistants, and the validation of the measurement instruments. Subsequently, each school was randomly allocated (cluster-level) to one of the study groups, and the informed consent process was carried out together with the application of the inclusion and exclusion criteria.

In Phase 2, pretest assessments were conducted, during which height, weight, and body mass index (BMI) were measured in accordance with the technical guidelines established by Resolution 2465 of 2016 of the Colombian Ministry of Health and Social Protection [15]. Selective attention was assessed using the d2-R test, and physical activity levels were evaluated using the Physical Activity Questionnaire for Children (PAQ-C).

Phase 3 corresponded to the eight-week intervention period across the different study groups and Phase 4 consisted of the posttest assessments, which were conducted under the same conditions as those applied during Phase 2.

### 2.4. Physical Activity Intervention

This study implemented a multimodal PA intervention designed to compare different PA intensities and pedagogical contexts over an eight-week period. The intervention was delivered by highly trained research assistants following standardized protocols to ensure consistency and reliability across the study groups. Across all four study groups, students continued receiving their regular PE classes as part of the curriculum with a weekly frequency. Additionally, the groups carried out the following activities as part of the study intervention.

CG participants maintained their habitual school routines, including regular PE classes, which were guided by each school’s curriculum. In both CG schools, they presented a frequency of one weekly class lasting 80 min. The CG PE classes were monitored by the researchers to identify in detail the activities developed in class, finding that the vast majority involved repetitive practices with moderate intensities and a focus on sports technique exercises.

EG 1 (MVPA). In this group, activities were conducted during physical education classes (90 min per session, once per week) and included sustained periods of moderate-to-vigorous intensity (≥7/10 on the EPInfant scale). Each session included a warm-up phase, and the main phase consisted of motor games, aerobic exercises, and sports-based activities while maintaining perceived exertion at high levels.

EG 2 (MPA). Activities in this group were implemented during school recess periods held at the midpoint of the daily school schedule (30 min per session, twice per week). The intervention consisted of structured moderate-intensity activities, primarily based on traditional games involving gross motor movements and conducted in the school’s outdoor spaces.

EG 3 (LPA). Activities were conducted during theoretical classes through light-intensity active breaks (10 min per session, three times per day, three days per week). These activities were designed to interrupt sedentary behavior without leaving the classroom. The sessions included rhythmic movements guided by the research assistant, general muscle stretching, coordination exercises, and traditional games such as *tingo*, *tingo*, *tango*.

In the intervention groups, previously trained research assistants recorded students’ perceived exertion at the end of each session using the EPInfant visual scale [16,17], in order to ensure that the level of effort corresponded to that assigned to each group.

### 2.5. Measures

All assessments were conducted using internationally validated instruments and coordinated by highly trained research assistants.

#### 2.5.1. Selective Attention: Main Cognitive Function

Selective attention was assessed using the Spanish adaptation of the d2-R attention test [18]. The test was administered in Week 1 (pre-intervention) and in Week 10 (post-intervention). Following the recommendations provided in the test manual, a controlled environment was prepared to ensure proper administration. The assessment was conducted using a paper-and-pencil format, and the data were subsequently entered into the scoring software platform to generate the test scores.

The d2-R Test (Revised) is an internationally validated cancellation task that is widely used in school settings. It is designed to assess the ability to focus on relevant stimuli while inhibiting distractors under time pressure. The test primarily yields two indicators: the percentage of errors relative to the total number of processed stimuli, reflecting attentional accuracy, and the concentration performance score, which indicates the level of selective attention [19].

#### 2.5.2. Control Variables

To account for potential confounding factors, data were collected on habitual PA, as well as sociodemographic and anthropometric control variables, across the four study groups. Habitual PA levels were assessed using the Physical Activity Questionnaire for Children (PAQ-C), which captures physical activity performed over the previous seven days across contexts such as leisure time, physical education classes, and school recess [20]. Sociodemographic variables (age, sex, and socioeconomic stratum) were collected during the completion of the written assessments. Anthropometric measurements were obtained by trained research assistants using a Tanita scale (MC-580; Tanita Corporation, Tokyo, Japan) to measure body weight and a portable stadiometer (SECA 206; Hamburg, Germany) aligned with the Frankfurt horizontal plane to measure height. Body mass index (BMI) was calculated in accordance with the recommendations of Resolution 2465 of 2016 issued by the Colombian Ministry of Health and Social Protection.

### 2.6. Statistical Analysis

All analyses were conducted in IBM SPSS Statistics, version 23.0 (institutional license, Universidad de La Sabana), using a significance threshold of *p* < 0.05 and 95% confidence intervals. Descriptive statistics were reported as means and standard deviations for continuous variables and as absolute frequencies and percentages for categorical variables.

Group comparisons of participant characteristics and baseline selective attention (d2-R) were performed using the Kruskal-Wallis test for all continuous variables due to violation of the normality assumption and the Chi-square test for categorical variables.

The primary analysis was performed using Linear Mixed Models (LMMs), given the longitudinal nature of repeated measures within each student (pretest and posttest). LMMs allow fixed and random effects to be incorporated simultaneously, enabling modelling of within-subject correlation and yielding appropriate estimates for dependent longitudinal data [21].

The fixed effects included Group (MVPA, MPA, LPA, Control), Time (pretest–posttest), and the Group × Time interaction, which represented the primary effect of interest for examining whether selective attention (d2-R) changes differed across physical activity dose conditions, which represented the primary study hypothesis. Baseline selective attention score, habitual physical activity level (PAQ-C score), and age were included as covariates given their significant between-group differences at baseline. A random intercept for participant (ID) was specified to account for individual variability, with a diagonal covariance structure for the repeated measures. Model parameters were estimated using Restricted Maximum Likelihood (REML).

The model assumptions were evaluated through diagnostic analyses of standardized residuals. Normality was examined by inspecting Q–Q plots, complemented by the Kolmogorov–Smirnov test, whereas homoscedasticity was assessed through visual inspection of residuals-versus-fitted values scatterplots. Although formal normality tests indicated minor deviations in the distribution tails, this pattern is common in large samples (*n* = 267) and does not compromise the inferential validity of LMMs, given their robustness in this context [22,23].

Pairwise comparisons of estimated marginal means (EMMs) were conducted across all groups, applying Bonferroni adjustment to control the type I error rate. Effect sizes were estimated using partial eta squared (η^2^*p*) for the overall Group × Time interaction, calculated using the formula η^2^*p* = (F × df_effect)/(F × df_effect + df_error) and interpreted according to Cohen: small (η^2^*p* = 0.01), medium (η^2^*p* = 0.06), and large (η^2^*p* = 0.14). Cohen’s d was calculated for pairwise post-hoc comparisons: trivial (<0.20), small (≥0.20), medium (≥0.50), and large (≥0.80).

## 3. Results

### 3.1. Participant Characteristics and Data Structure

A total of 267 children and adolescents from eight schools in Bogotá, Colombia, were included in the final analysis and distributed into four groups: control (CG; *n* = 69, 25.8%), light-intensity physical activity (LPA; *n* = 64, 24.0%), moderate-intensity physical activity (MPA; *n* = 66, 24.7%), and moderate-to-vigorous physical activity (MVPA; *n* = 68, 25.5%). As shown in Table 1, the mean age of the sample was 10.42 ± 1.11 years, with a predominance of male participants (57.7%). Significant differences between groups were observed for age, body weight, and height (all *p* < 0.001), while sex distribution was comparable across groups (*p* = 0.478); therefore, age was included as a covariate in the Linear Mixed Model. Regarding BMI, most participants presented adequate weight for age (56.9%), followed by overweight (28.1%) and obesity (8.6%), with significant differences in BMI distribution across groups (*p* = 0.001), particularly in the LPA group, which showed the highest prevalence of obesity (17.2%), and the MVPA group, which presented the highest prevalence of overweight (38.2%) Table 1.

As measured by the PAQ-C [24,25,26], significant differences in physical activity levels were observed across groups (*p* < 0.001). The majority of participants were classified in the moderately low PA category (58.8%), followed by low PA (34.5%) and moderate PA (6.7%), with no students reaching the moderately high or high PA categories [27]. When stratified by sex, girls showed lower physical activity levels than boys, with a higher proportion classified as low PA (38.1% vs. 31.8%), moderately low PA (55.8% vs. 61.0%), and moderate PA (6.2% vs. 7.1%), consistent with reports from the Active Healthy Kids Global Alliance for Colombia, which describe lower physical activity levels in girls compared with boys of the same age [3].

### 3.2. Baseline Characteristics of Selective Attention (d2-R)

Prior to the intervention, a statistically significant difference in selective attention was observed across groups (Kruskal-Wallis test; *H*(3) = 11.34, *p* = 0.010). Therefore, the pre-intervention score was included as a covariate in the Linear Mixed Model to control for its potential influence on post-intervention outcomes.

### 3.3. Selective Attention (d2-R)

Within-group pre-to-post comparisons revealed significant improvements in all intervention groups (MVPA: Δ = 17.49, *p* < 0.001; LPA: Δ = 15.47, *p* < 0.001; MPA: Δ = 9.41, *p* = 0.001), while the control group showed no significant change (Δ = 2.51, *p* = 0.341; Table 2).

A statistically significant Group × Time interaction was found (*F*(3, 263) = 17.92, *p* < 0.001, η^2^*p* = 0.170), indicating a large differential effect of the physical activity interventions on selective attention.

**Figure 4:**
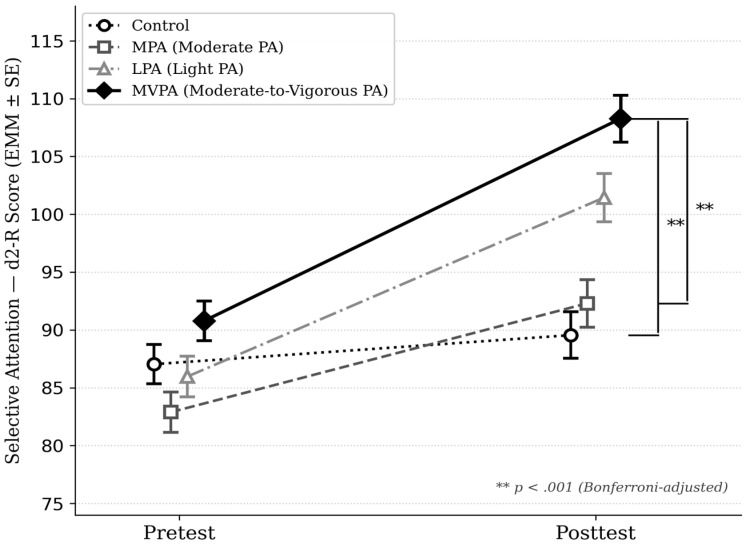
Estimated marginal means of selective attention (d2-R) by group and time.

Figure 4 illustrates the divergent trajectories derived from estimated marginal means (EMMs), highlighting a considerably steeper slope in the MVPA group relative to MPA and CG. Likewise, an upward trend is visible for the LPA group, although this progression did not meet the threshold for statistical significance under the model’s parameters. To facilitate the interpretation of these findings, brackets delineate significant inter-group differences, which were validated through Bonferroni adjustment (** *p* < 0.001). Both means and standard errors (±1 SE) were adjusted for baseline attention scores, age, and prior PA levels.

Post-hoc pairwise comparisons with Bonferroni correction (Table 3) revealed that the MVPA group significantly outperformed both the control group (MD = 11.23, 95% CI [4.88, 17.57], *p* < 0.001, *d* = 1.08, large effect) and the MPA group (MD = 11.92, 95% CI [5.51, 18.34], *p* < 0.001, *d* = 0.88, large effect). No significant difference was found between MVPA and LPA (*p* = 0.105). The LPA group did not differ significantly from the control group (*p* = 0.160) or MPA (*p* = 0.080), and no significant difference was observed between MPA and controls (*p* = 1.000).

These findings suggest a response pattern in which selective attention improvements were greatest in the MVPA group, with no significant differential effect observed for MPA or LPA relative to the control group after Bonferroni adjustment.

## 4. Discussion

### 4.1. Synthesis of Findings in Relation to the Study Questions

The present study was designed to evaluate and compare the effects of a multimodal PA program on selective attention in schoolchildren aged 8–14 years. An eight-week intervention was implemented simultaneously across four study groups, each receiving a different stimulus according to the prescribed physical activity intensity. Our main findings indicate that selective attention increased significantly in the three intervention groups, comparing pre and post intervention scores; however, the benefits did not follow a linear distribution that was directly proportional to increasing intensity. Specifically, the MVPA group showed significant improvement, followed by the LPA group, while the MPA did not show large increases in selective attention scores.

For comparisons between study groups, the MVPA implementation produced higher selective attention values than the control, LPA, and MPA groups. The LPA and MPA interventions showed higher scores than the control group, but the difference was not significant.

In relation to the above, these findings address the primary question of this study by showing that PA practice is associated with benefits in selective attention; this phenomenon aligns with previous literature highlighting that those practices combining physical demands with motor challenges (agility, coordination, sport) favor this cognitive function [14,28,29]. Regarding the second question addressed by the present study, the results indicate that MVPA may yield the greatest benefits for selective attention, at least under the intervention and evaluation approach used in this research.

### 4.2. Neurophysiological Mechanisms Associated with MVPA

A possible explanation for why MVPA produced greater benefits on selective attention can be found in numerous recent publications that describe the relationship between PA, especially MVPA, and neurophysiological mechanisms derived from exercise practice. These physiological responses can be grouped into three main domains: biochemical and neurotrophic mechanisms, neural activation and efficiency mechanisms, and structural and systemic mechanisms. Within biochemical and neurotrophic mechanisms, effects such as increases in plasma levels of brain-derived neurotrophic factor (BDNF), irisin, and lactate have been described, which appear to reach brain areas where they promote synapse formation and, eventually, new neurons. This has been associated with benefits in academic performance [30,31], whereas interventions involving moderate-intensity efforts (MPA) have not shown similar effects [32].

In the neural activation and efficiency domain, studies using techniques such as functional near-infrared spectroscopy (fNIRS) have shown improvements in cognitive functions associated with MVPA, together with changes in perfusion and, therefore, activation of the prefrontal region that are compatible with greater neural efficiency across different tasks, including learning and cognition [33]. In the structural domain, empirical evidence has reported associations between MVPA and brain morphology, suggesting greater neuronal and synaptic density in areas that are fundamental for memory [34,35].

### 4.3. Findings for the MPA Group and Change in the Control Group

A relevant aspect of the study findings was the lack of statistical significance for the MPA group compared with the CG (*p* = 1.000). Although MPA showed a nominal improvement (+9.41 points), it was not sufficient to meet the stringency of the Bonferroni adjustment. One possible explanation relates to the weekly intervention dose (two 30 min sessions = 60 min) compared with the other two intervention groups (90 min), as well as the implementation setting (school recess), which may have limited the stimulus and prevented sufficient adaptations. This non-linear response in relation to physical activity intensity has been discussed in recent studies, suggesting that the dose–response relationship is more complex than proposed by traditional models and may vary according to intensity, age, and the type of stimulus [36,37].

The increase in selective attention scores in the control group was very small (2.51 points), which could be explained by what is referred to in the scientific literature as practice effects due to repetition or task familiarization. This phenomenon is frequently observed in pretest–posttest assessments using paper-and-pencil tools for the evaluation of cognitive functions, where improvements at retesting may occur even in the absence of specific interventions [38,39].

### 4.4. Practical Implications in School Settings

The practical implications of this study go beyond the experimental scope and offer an opportunity to call the attention of school leadership, teachers, and educational public policies to redesign traditional curricula, allowing prolonged sedentary behaviors to be reduced and more active school activities to be integrated, while recognizing the potential of PA and the benefits of its practice in strengthening selective attention as a cognitive function linked to learning processes and academic performance.

### 4.5. Interpretation of Classroom Active Breaks (LPA)

An important finding that deserves special attention is the performance of the active breaks group (LPA), which achieved a substantial score in selective attention (Δ = +15.47), positioning itself above the CG and MPA in the nominal scores. Despite being a low-intensity stimulus, its results could be related to several factors. On the one hand, breaks from sedentary behavior appear to elevate the state of physiological arousal toward superior attention levels, especially when interspersing movement within cognitively demanding theoretical blocks [40].

On the other hand, the protocol designed with short sessions applied recurrently during the day ensures an accumulated motor exposure that favors the accumulation of PA throughout the week. From an applied perspective, the feasibility of these active breaks in the classroom is total, initially because they are highly adaptable and require a minimal investment of resources, dispensing with sports facilities or complex adjustments in the school schedule. This latter characteristic takes on a strategic value in contexts where infrastructure is limited or in populations with high levels of sedentary behaviors. In fact, given that almost the entire population presented low or moderately low PA levels, this type of intervention integrated into the classroom constitutes a practical and efficient tool to enhance cognitive benefits in educational environments.

### 4.6. Strengths and Limitations

The main strengths of this study include the sample size (*n* = 267) within a school setting; the use of LMMs for statistical analysis, which allowed the longitudinal nature of the data and baseline differences to be handled rigorously; the use of standardized and internationally validated instruments such as the d2-R; and the multimodal comparison of physical activity interventions including a control group. The main limitations are related to convenience sampling of schools and cluster-level (school) allocation, given the use of convenience sampling, and the absence of devices such as accelerometers or heart rate monitors for objective monitoring of PA.

Future research is recommended to incorporate measures that allow the long-term behavior of selective attention to be examined (Chronic effects); include populations diagnosed with attention deficit hyperactivity disorder (ADHD), for whom selective attention represents a core difficulty; and implement interventions that also consider other cognitive functions that may benefit from physical activity.

## 5. Conclusions

The results of this research provide robust evidence on the effect of an eight-week multimodal physical activity program on selective attention in schoolchildren aged 8–14 years. These findings allow us to recommend the structured integration of physical activity programs within the school curriculum as a strategy to strengthen attentional processes and potentially impact academic performance.

In response to our first research question, the results suggest that physical activity in school settings may contribute to improvements in selective attention as a cognitive function. Regarding the second research question, the findings point to a non-linear relationship between the stimulus dose by intensity and the response in selective attention, with MVPA showing the greatest benefits, followed by LPA, which reports significant increases in the nominal scores, while MPA did not present significant differences compared with the CG.

## Figures and Tables

**Table 1:** Participant Characteristics.

Variable	Total	Control	LPA	MPA	MVPA	*p*
	(*n* = 267)	(*n* = 69)	(*n* = 64)	(*n* = 66)	(*n* = 68)	
**Age (years)**	10.42 ± 1.11	10.01 ± 0.93	11.47 ± 1.09	10.47 ± 0.82	9.81 ± 0.81	<0.001
**Sex**						0.478
Female	113 (42.3)	26 (37.7)	27 (42.2)	26 (39.4)	34 (50)	
Male	154 (57.7)	43 (62.3)	37 (57.8)	40 (60.6)	34 (50)	
**Body weight (kg)**	38.20 ± 9.31	35.43 ± 6.73	43.04 ± 12.24	35.64 ± 8.16	38.95 ± 7.42	<0.001
**Height (cm)**	1.41 ± 0.08	1.37 ± 0.06	1.46 ± 0.08	1.40 ± 0.07	1.42 ± 0.08	<0.001
**BMI**						0.001
Obesity	23 (8.6)	6 (8.7)	11 (17.2)	4 (6.1)	2 (2.9)	
Overweight	75 (28.1)	24 (34.8)	13 (20.3)	12 (18.2)	26 (38.2)	
Adequate for age	152 (56.9)	36 (52.2)	38 (59.4)	39 (59.1)	39 (57.4)	
Risk of thinness	15 (5.6)	3 (4.3)	2 (3.1)	9 (13.6)	1 (1.5)	
Thinness	2 (0.7)	0 (0.0)	0 (0.0)	2 (3.0)	0 (0.0)	
**Physical Activity (PAQ-C)**						<0.001
Low	92 (34.5)	33 (47.8)	29 (45.3)	20 (30.3)	10 (14.7)	
Moderately low	157 (58.8)	35 (50.7)	34 (53.1)	41 (62.1)	47 (69.1)	
Moderate	18 (6.7)	1 (1.4)	1 (1.6)	5 (7.6)	11 (16.2)	
**PAQ-C Score**	2.19 ± 0.50	2.02 ± 0.40	2.01 ± 0.44	2.24 ± 0.50	2.50 ± 0.49	<0.001

Note: Continuous variables are expressed as mean ± standard deviation and compared between groups using the Kruskal-Wallis test due to violation of the normality assumption. Categorical variables are expressed as frequency (*n* and %) and compared using the Chi-square test.

**Table 2:** Estimated marginal means (EMMs) of d2-R attention by group and time.

Group	Pre-EMM [95% CI]	Post-EMM (95% CI)	Δ	*p*
Control	87.04 (83.71–90.38)	89.55 (85.59–93.51)	2.51	0.341
LPA	85.97 (82.51–89.43)	101.44 (97.33–105.55)	15.47	<0.001
MPA	82.89 (79.48–86.30)	92.30 (88.25–96.35)	9.41	0.001
MVPA	90.78 (87.42–94.14)	108.27 (104.28–112.26)	17.49	<0.001

Note: EMM = estimated marginal mean adjusted for baseline selective attention score, physical activity level, and age. CI = confidence interval. Δ = post-pre change. *p* = within-group pre-to-post comparison (Bonferroni-adjusted). Group × Time interaction: *F*(3, 263) = 17.92, *p* < 0.001, η^2^*p* = 0.170. Effect size magnitude classified according to Cohen (1988): small ≥ 0.01, medium ≥ 0.06, large ≥ 0.14.

**Table 3:** Post-hoc Pairwise Comparisons of Selective Attention Between Groups.

Comparison	MD	95% CI	*p*	*d*	Magnitude
MVPA vs. Control	11.23	[4.88, 17.57]	<0.001	1.08	Large
MPA vs. Control	−0.70	[−7.09, 5.70]	1.000	0.18	Trivial
LPA vs. Control	5.41	[−1.04, 11.85]	0.160	0.79	Medium
MVPA vs. MPA	11.92	[5.51, 18.34]	<0.001	0.88	Large
MVPA vs. LPA	5.82	[−0.65, 12.29]	0.105	0.39	Small
LPA vs. MPA	6.11	[−0.41, 12.62]	0.080	0.57	Medium

Note: MD = mean difference based on estimated marginal means. *p*-value-adjusted Bonferroni correction. CI = confidence interval. *d* = Cohen’s d: trivial < 0.20, small ≥ 0.20, medium ≥ 0.50, large ≥ 0.80.

## Data Availability

The data supporting the findings of this study are available from the corresponding author upon reasonable request in order to protect the privacy of the minors who participated in this study.

## Abbreviations

The following abbreviations are used in this manuscript:

ADHD	Attention Deficit Hyperactivity Disorder
BDNF	Brain-Derived Neurotrophic Factor
BMI	Body Mass Index
CG	Control Group
d2-R	Attention test d2 Revised
EG	Experimental Group
EMM	Estimated Marginal Mean
EPInfant	Escala de Medición de Esfuerzo Percibido Infantil
fNIRS	Functional Near-Infrared Spectroscopy
LPA	Light Physical Activity
LMM	Linear Mixed Model
MVPA	Moderate-to-Vigorous Physical Activity
MPA	Moderate Physical Activity
PA	Physical Activity
PAQ-C	Physical Activity Questionnaire for Older Children
REML	Restricted Maximum Likelihood
SPSS	Statistical Package for Social Sciences
